# Eating disorders mothers and their children: a systematic review of the literature

**DOI:** 10.1007/s00737-020-01019-x

**Published:** 2020-01-14

**Authors:** Maria Giulia Martini, Manuela Barona-Martinez, Nadia Micali

**Affiliations:** 1 Behavioural and Brain Sciences Unit, Institute of Child Health, University College London, 4th Floor, 30 Guilford Street, London, WC1N 1EH UK; 2 South London and Maudsley, NHS Foundation Trust, London, UK; 3 Department of Psychiatry, University of Geneva, Geneva, Switzerland; 4 Child and Adolescent Psychiatry Division, Department of Child and Adolescent Health, University Hospital Geneva, Geneva, Switzerland

**Keywords:** Eating disorder, Intergenerational effect, Children, Mothers

## Abstract

To provide an overview of the impact of maternal eating disorders (ED) on child development in a number of domains including feeding and eating behaviour, neuropsychological profile and cognitive development, psychopathology and temperament. PubMed, Embase and PsychInfo were searched for studies exploring the impact of maternal ED on children between January 1980 and September 2018. Initial search yielded 569 studies. After exclusion, 32 studies were reviewed. Overall, available evidence shows that children of mothers with ED are at increased risk of disturbances in several domains. They exhibit more difficulties in feeding and eating behaviours, display more psychopathological and socio-emotional difficulties, and they are more likely to be described as having a difficult temperament. Maternal ED have an impact on child psychological, cognitive and eating behaviours, and might affect the development of ED in the offspring. Future research should focus on resilience and on which protective factors might lead to positive outcomes. These factors can be then used as therapeutic and preventative targets.

## Introduction

Eating disorders (ED) are mental health disorders characterised by severe disturbances in eating behaviour that significantly impact an individual’s emotional, psychosocial and physical well-being (Bannatyne et al. 2018). Current diagnostic classifications of ED include anorexia nervosa (AN), bulimia nervosa (BN) and binge-eating disorder (BED) as full threshold ED. ED typically affect women of reproductive age (Easter et al. 2014).

Early childhood is a crucial time for the development of the mother–child relationship. It is during this time that children develop psychosocially, engage in social learning and express their temperament—which is a biological tendency within each child but it is shaped by complex interactions between genetic, biological and environmental factors (Shiner et al. 2012).

Risk for developmental problems in children of women with psychiatric disorder has been well documented in literature and several aspects of children’s development can be affected, including their physical, cognitive, social, emotional and behavioural development (Ramchandani and Stein 2003). Fewer studies are available regarding the impact of ED on child development.

However, the literature available suggests that children of mothers with ED have an increased risk for negative developmental outcomes, including cognitive, social and emotional disturbances (Patel et al. 2002). Research has shown that children of mothers with ED are more likely to develop an emotional disorder at the age of 7 and 10 (Micali et al. 2014a), they are more likely to show neurobehavioural dysregulation early after birth and poorer language and motor development at 1 year (Barona et al. 2017).

Furthermore, there is evidence that children of mothers with ED are more likely to develop ED themselves (Kothari et al. 2013).

Family and twin studies have consistently demonstrated that ED have a strong genetic component (Mazzeo et al. 2005); however, the environment is likely to play a crucial role in the expression of underlying genetic predispositions.

In 2005, Bulik et al. (2005) proposed a cycle of risk model for the development of AN, whereby the maternal effect of AN on their children via perinatal complications is hypothesised as being influenced by environmental factors, genetic factors and environmental factors that are highly influenced by maternal genotype (i.e. pregnancy nutrition, weight gain in utero, appearance focus and restrictive eating during childhood/adolescence). In 2009, Micali and Treasure suggested a risk model for the impact of maternal ED on child development that embraces all ED focusing on in utero mechanisms. In particular, the model explained the effect of a maternal ED in pregnancy on the foetus via nutritional factors (including protein deficiency, low folate and low iron intake) and comorbid psychopathology (i.e. comorbid anxiety and depression and in turn via increased glucocorticoids and corticotrophin-releasing hormone) which both could lead to obstetric complications (Micali and Treasure 2009). The influence of parental ED on child phenotype might likely be the result of a complex interplay between genetic (maternal and child) and environmental factors.

Understanding the mechanisms leading from maternal ED to adverse child development could help to determine both risk and protective factors that could be potentially targeted for intervention.

To date, only one recent systematic review has been published on this topic in the last several years and the authors did focus only on the most recent findings (2015 onwards) (Watson et al. 2018). Previous reviews focusing on children of mothers with ED include (Patel et al. 2002) and (Park et al. 2003). No systematic reviews have been carried out so far covering the period between 2003 and 2015 and many relevant population-based studies have been carried out over this period.

The purpose of our paper is to provide an overview of the impact of maternal ED on child development. Particularly, this review will focus on effects on developmental aspects, across the spectrum of feeding and eating behaviour, neuropsychological profile and cognitive development, psychopathology and temperament.

## Methods and materials

### Data source

A systematic and comprehensive search of databases, including PsychInfo, Embase and Medline, was carried out for studies published between January 1980 and September 2018. The search was performed using the following mesh terms and keywords: (‘maternal eating disorders’ or ‘maternal anorexia nervosa’ or ‘maternal bulimia nervosa’ or ‘maternal binge eating disorder’ or ‘mothers with eating disorders’ or ‘mothers with anorexia nervosa’ or ‘mothers with bulimia nervosa’ or ‘mothers with binge eating disorder’) and (‘child development’ or ‘child language development’ or ‘child feeding’ or ‘child cognitive development’ or ‘child temperament’ or ‘child psychopathology’).

### Study selection

Inclusion criteria for the studies included (1) exposure (mothers) diagnosed with any ED (i.e. AN, BN and BED) either active or past, (2) the outcome was a measure of child development assessed from birth up until 12 years of age, (3) studies published in English and (4) primary studies.

Exclusion criteria for the studies included the following: studies were excluded if the aims were to investigate child eating pathology. Papers were also excluded if an intervention was assessed such as video-feedback interactional treatment.

### Quality assessment and data extraction

Two authors (M.G.M. and M.B.) independently screened, extracted and cross-checked the data based on a priori exclusion and inclusion criteria. The quality of the final studies was also independently checked by both authors using the Newcastle–Ottawa Scale (NOS) for assessing the quality of non-randomised studies in meta-analyses (Table 1). The above scale includes case–control and cohort studies.

**Table 1:** Studies exploring the impact of maternal Eating Disorder (ED) on child development

Authors	Study, design	Participants: *n*, age (SD), diagnoses and recruitment	Measures (exposure and outcome)	Results	NOS rating
FEEDING/EATING					
1 Feeding/Eating Stein et al. 1994	Case–control	Total *n* = 58 mothers Cases recruited from community *n* = 34; mean age 28.3 12 EDNOS, 6 BN and 16 subthreshold Healthy controls recruited from community *n* = 24; mean age 29.0	Exposure: Maternal ED (Clinical Interview through Eating Disorder Examination—EDE) Outcome: infant development: Bayley Scales feeding and growth: Tanner and Whitehouse’s specification	▪ *Negative expressed emotion* was more frequent among the case mothers compared to controls during mealtimes but *not* during play ▪ Mothers with ED were less facilitating during both mealtimes and play, had significantly more conflict with infants ▪ Case infants were rated as less happy than the controls during both mealtime and play	9/9
2 Feeding/Eating/Temperament Evans and le Grange 1995	Case–control	Total *n* = 20 Cases, AN and BN *N* = 10, mean age = 36.0 (7.02) Healthy controls *N* = 10, mean age = 35.4 (4.12)	Exposure: Maternal ED (Clinical Interview DSM III-R APA) Outcome: body shape questionnaire; semi-structured interview on feeding and development	▪ Positive correlation found in both case and control groups between mothers’ satisfaction with their body size and their children’s satisfaction with their own weight and shape ▪ Mothers in the clinical group reported experiencing emotional problems when breast feeding their children ▪ Infants in the clinical group were schedule fed—this rigid adherence caused some confusion and anxiety for mothers when their infants displayed signs of hunger outside the recommended feeding times ▪ Half the children of ED mothers were described as displaying difficulties such as hyperactivity, avoidant behaviour, enuresis, insecure attachment, depression, fears, personality problems, stuttering, violent temperament and oppositional defiant behaviour	8/9
3 Feeding/Eating/Temperament Agras et al. 1999	Case–control	Total *n* = 194 Cases *N* = 41, mean age = 32.1 (4.4) AN = 2, BN = 17, BED = 22 Healthy controls *N* = 153, mean age 32.9 (3.8)	Exposure: maternal ED (Clinical Interview DSM III-R + Eating Disorder Inventory—EDI) Outcome: Infant Feeding Report (IFR); Suckometer; Children’s Behaviour Questionnaire	▪ Female infants of ED mothers sucked more rapidly than other infants, but no differences in caloric intake at these feedings ▪ ED mothers bottle fed their daughters for a mean time of 33.2 months compared with infants of NED mothers with 23.6 months ▪ ED mothers considered their female children to have more difficulty in weaning from the bottle (but not from the breast) than NED mothers ▪ Infants of ED mothers were reported to dawdle more while eating compared to children of NED group ▪ ED mothers reported their daughters as vomiting more frequently than their sons (opposite effect for NED mothers) ▪ ED mothers reported higher concern for their daughter’s weight than NED mothers ▪ Significant main effect for the ED group for using food as non-nutritive purposes ▪ Significant effect of ED mothers reporting they fed their children on a less regular schedule than NED mothers ▪ Children of mothers with ED reported as demonstrating more negative affect (sadness, crying, irritability) than children of NED mothers	8/9
4 Feeding/Eating Stein et al. 1999	Case–control	Total *n* = 58 Cases *N* = 34, mean age = 28.3 Healthy controls *N* = 24, mean age = 29	Exposure: maternal ED (Clinical Interview DSM III R) Outcome: Five-Point Conflict/Harmony Scale	▪ The most frequent antecedent to conflict was the mother’s concern about the manner of eating; disagreement over who fed the infant and food refusal ▪ Mothers in the clinical group only acknowledged the infant’s signals in a third of cases compared to over a half in the NED mothers group	5/9
5 Feeding/Eating Temperament Waugh and Bulik 1999	Case–control	Total *n* = 20 mothers *N* = 10, mean age 30.1 (3.1) Past AN = 6, past BN = 7 Healthy controls recruited from community *n* = 10, mean age 30.8 (3.6)	Exposure: maternal ED (Clinical Interview DSM III-R) Outcome: Toddler Temperament Scale (TTS); Mealtime Observation Schedule (MOS); Food Diary	▪ Children of women with eating disorders had significantly lower birth weights and lengths than control children ▪ No differences observed in childhood temperament or mothers’ satisfaction with children’s appearance ▪ Mothers with ED made significantly fewer positive eating comments	8/9
6 Feeding/Eating Whelan and Cooper 2000	Case–control	Total *n* = 128 mothers Clinical group (any ED disorder) Children split in three groups: ▪ Clinical—feeding problems (1): 42 ▪ Clinical—disturbed comparisons (2): 79 ▪ Control: 29	Exposure: maternal current and past affective disorder and current and past ED (Eating Disorder Examination—EDE + Clinical Interview DSM IV) Outcome: Shyness; Preschool Behaviour Checklist (PBCL); Behaviour Screening Questionnaire (BSQ); Feeding Problems and Eating Disorders Interview Schedule	▪ Children in the feeding problem group were rated as significantly more disturbed than the control group (feeding disturbances such as refusal, faddiness and spitting) ▪ Severity of child disturbance was not related to the relationship between feeding problems and maternal ED	8/9
7 Feeding/Eating Blissett and Meyer 2006	Case–control	Total *n* = 114 mothers and 114 children Mean maternal age 33 (5.5) Mean child age 29 months (13.77)	Exposure: Maternal ED (Eating Disorder Inventory 2—EDE-2 questionnaire) Outcome: Child Feeding Assessment Questionnaire (CFAQ); Young Schema Questionnaire	▪ Eating psychopathology did not explain mealtime negativity in boys ▪ Eating psychopathology failed to explain mealtime negativity in girls Food refusal ▪ Eating psychopathology failed to explain food refusal in boys Eating psychopathology added significantly to the variance explained in food refusal of girls	6/9
8 Feeding/Eating Micali et al. 2009	Cohort	Total *n* = 12,050 ALSPAC Cases *N* = 441, AN = 247 and BN = 194 Mean age, AN = 29.1 (5.0), BN = 28.3 (4.6) Healthy controls *N* = 10,461, mean age = 28.2 (4.8)	Exposure: Maternal ED (self-report) Outcome: feeding questionnaires; health records	▪ Mothers with a history of ED were more likely to start breast feeding than controls (83% with 76%) ▪ Also less likely to stop breast feeding during the first year of infant life ▪ Those with BN were more likely to continue breast feeding Infant feeding ▪ AN mothers reported more early onset persistent feeding difficulties in all domains except refusal to take solids ▪ Infants of mothers with BN differed in the rate of refusal to take solids from those AN mothers in the rates of being unsatisfied/hungry after feeding	7/9
9 Psychopathology/Feeding/Eating Reba-Harrelson et al. 2010	Cohort	Total *n* = 13,006 MoBa (Norwegian mothers and child cohort study) Cases *N* = 479, BED = 634, BN = 98, AN = 17 Healthy controls *N* = 12,257	Exposure: maternal ED (self-report) Outcome: Child Feeding Questionnaire; Child Behaviour Checklist (CBCL); Infant Toddler Social Emotional Assessment (ITSEA)	▪ Mothers with BN and BED reported higher levels of disordered eating behaviours in their children than controls ▪ They also reported higher levels of anxiety symptoms in their children ▪ Mothers with BN reported higher levels of OCD symptoms in their children ▪ Maternally reported restrictive feeding was significantly associated with child disordered eating difficulties	8/9
10 Feeding/Temperament Micali et al. 2011	Cohort	Total n = 10,902 ALSPAC Cases *N* = 441, AN = 166, BN = 194, AN + BN = 81; mean age = 28.7 (4.8) Healthy controls *n* = 10,461; mean age = 28.2 (4.8)	Exposure: maternal ED (self-report), maternal depression, maternal anxiety Outcome: maternal reports; Carey Infant Temperament Scale; Denver Developmental Scale	▪ Maternal lifetime ED predicted feeding difficulties at 1 month and 6 months ▪ ED symptoms in pregnancy found to predict feeding difficulties at 1 month ▪ Child ‘difficult’ temperament score was associated with late feeding difficulties	8/9
11 Feeding/Eating Easter et al. 2013	Cohort	Total *n* = 9423 ALSPAC Cases *N* = 387, AN = 140, mean age = 29.7 (5.2), BN = 170, mean age = 28.3 (4.6), AN + BN = 71, mean age = 29.6 (4.6) Healthy controls *N* = 9037, mean age = 28.8 (4.8)	Exposure: maternal ED (self-reported) Outcome: Food Frequency Questionnaire (FFQs); nutritional intake	▪ Children of mothers with all 3 ED groups (AN, BN and AN + BN) had higher scores on the health conscious/vegetarian dietary pattern across all 4 time points: 3, 4, 7 and 9 years* ▪ Differences persisted in maternal AN and BN groups after adjustments—children scored lower on the traditional dietary pattern across all 4 time points ▪ Trends showed higher energy intake of children with mothers with BN and AN + BN ▪ Children with mothers with BN had higher starch intake	8/9
12 Feeding/Eating Hoffman et al. 2014	Cohort	Total *n* = 50 Recruited from community Cases *N* = 25; AN = 13; BN = 13; EDNOS = 2, mean age = 32.7 (4.61) Healthy controls = 25, mean age 29.68 (1.99)	Exposure: maternal ED (clinical interview SCID-I) Outcome: anthropometric data/infant feeding style questionnaire/Toddler Diet Questionnaire/EDE-Q/BDI/BAI	▪ Mothers with histories of eating disorders scored significantly lower on the restrictive feeding style subscale than control mothers ▪ No significant differences between groups in child diet (i.e. duration of breast feeding, age at solid food introduction, daily number of snacks and meals etc) ▪ Mothers with eating disorder histories were more likely to report taking a restrictive special approach to feeding such as limiting processed foods or feeding organic foods only	7/9
13 Feeding/Eating de Barse et al. 2015	Cohort	Total *n* = 4851 Generation R study Cases *N* = 415, mean age = 30.8 (5.0) AN = 121, BN = 189, AN + BN = 105 Healthy controls *N* = 4436, mean age = 30.8 (4.8)	Exposure: maternal ED (self-report questionnaire) Outcome: Child Feeding Questionnaire (CFQ); Child Eating Behaviour Questionnaire (CEBQ); Children’s body mass index	▪ Mothers with a history of ED used less pressure to eat than mothers without ▪ Mothers with a history of AN were likely to use low levels of pressure to eat ▪ Children of mothers with an ED had higher levels of emotional overeating than controls—this was strongest with mothers with a history of AN	8/9
14 Feeding/Eating Torgersen et al. 2015	Cohort	Total *n* = 49,045, mean age = 29.6 (4.6) MoBa (Norwegian mothers and child cohort study) Cases *N* = 3013 AN = 44, BN = 436, BED = 2475, EDNOS–P = 58 Healthy controls *N* = 46,032	Exposure: maternal ED (Self-reported) questionnaire Outcome: Infant Diet Questionnaire 4	▪ Percentages of mothers breast feeding at 6 months: BN (79%), BED (76%), EDNOS-P (59%), AN (58%), no-ED (82%) ▪ Infants of mothers with BN had significantly lower odds of being in the *homemade traditional food* class than the *commercial jarred baby food* class ▪ Infants of mothers with BED were significantly less likely to be in the *homemade vegetarian food* class than the *commercial jarred baby food*	7/9
15 Feeding/Eating Saltzman et al. 2016	Case–control	Total *n* = 260 Cases *N* = 36 mothers with BED Healthy controls *N* = 224	Exposure: frequency of BED behaviours through self-report questionnaire Eating Disorder Diagnostic Scale (EDDS) Exposure: Children’s Negative Emotions Scale; Comprehensive Feeding Practices Questionnaire; Child BMI	▪ Maternal BED predicted use of more nonresponsive feeding practices (e.g. Emotion Regulation, Restriction for Health, Pressure to Eat, and Food as Reward) ▪ Maternal BED was associated with greater use of distress responses, which indirectly predicted higher child BMI percentile through Food as Reward feeding practices	5/9
16 Feeding/Eating Nguyen et al. 2017	Cohort	Total *n* = 6196 Generation R study Cases *N* = 591 Healthy controls *N* = 5605	Exposure: self-report questionnaire Outcome: Food Frequency Questionnaire (FFQ), breastfeeding duration	▪ Mothers with a history of EDs seem slightly less likely to initiate breast feeding, although no longer statistically significant after adjustment ▪ At the age of 1 year, infants of mothers with a history of EDs had a higher diet quality	7/9
17 Feeding/Eating Martini et al. 2018	Case–control	Total *n* = 99 Cases *N* = 53; past ED 28, mean age = 33.3 (5.5); current ED 25, mean age = 29.0 (5.4) Healthy controls *N* = 46, mean age = 34.0 (3.8)	Exposure: clinical interview (SCID-I) Outcome: Infant Feeding Questionnaire (IFQ)/Parental Modelling of Eating Behaviours Scale (PARM)	▪ Women with P-ED and C-ED reported higher concerns about their infant being/becoming overweight compared with HC, respectively, at 8 weeks and 6 months and 6 months only post partum ▪ Women with P-ED showed less awareness of infant hunger and satiety cues compared with HC at 8 weeks	8/9
COGNITIVE DEVELOPMENT/NEUROPSYCHOLOGICAL PROFILE					
1 Cognitive development/Neuropsychological profile Kothari et al. 2014	Cohort	Different sample sizes were taken into account Total *n* = 982/852 Healthy controls: 937/819 Cases AN = 14; BN = 18; AN + BN = 13 AN = 11; BN = 12; AN and BN = 10	Exposure: self-report questionnaire Outcome: Griffiths Development Scales; Wechsler Preschool and Primary Scale of Intelligence–Revised	Griffiths development scales ▪ Children of women with a lifetime of AN showed lower scores on the development of locomotor and personal–social development than children of unexposed mothers Wechsler Preschool and Primary Scale of Intelligence–Revised ▪ Children of women with a lifetime of AN showed lower scores on performance subtests of geometric design and block design ▪ They also showed lower scores on vernal tests of comprehension and similarities They also showed lower verbal IQ scores in comparison to the controls	7/9
2 Cognitive development/Neuropsychological profile Kothari et al. 2013	Cohort	Total *n* = 11,088 Cases = 446 Mean age = 29.17 (4.49) AN = 194, BN = 171 and AN + BN = 81 Healthy controls *N* = 10,642, mean age = 29.10 (4.5)	Exposure: maternal ED (self-report) questionnaire Outcome: Wechsler Intelligence Scale for Children (WISC-III); Tests for Everyday Attention for Children; Counting Span Task; Stop-Signal Paradigm	Intelligence and global cognition ▪ Children of AN mothers showed higher full-scale IQ and performance IQ than compared with NED mothers ▪ Children of AN mothers showed high picture arrangement scores on the WISC subtest whereas children of BN mothers showed low object assembly scores Working memory (WM) ▪ Children of AN mothers showed slightly better WM span scores after adjustments ▪ Children of AN + BN mothers showed better global WM scores	7/9
3 Cognitive development/Neuropsychological profile Koubaa et al. 2013	Cohort	Total *n* = 112 Cases *N* = 47; AN = 24; BN = 20; EDNOS = 3, mean age = 29.3 (4.6) Healthy controls = 65, mean age 30 (3.7)	Exposure: maternal ED (clinical interview) Outcome: anthropometric measurement/Maternal Adjustment and Maternal Attitude Questionnaire (MAMA)/Five to Fifteen (FTF)	▪ Children born to mothers with ED had a lower birth weight but displayed an early catch-up in weight ▪ The average head circumference was found to be delayed up to at least 18 months of age ▪ Children of mothers with AN or BN had significantly higher FTF scores than controls reflecting difficulties in language and social skills	8/9
4 Cognitive development/Neuropsychological profile Kothari et al. 2015	Cohort	Total *n* = 1128 Cases *N* = 266 AN–R = 58 AN–BP = 66 BED = 72 Purge = 70 Healthy controls *N* = 862	Exposure: clinical interview (SCID) Outcome: Social Communication Disorders Checklist (SCDC); Diagnostic Analysis of Non-Verbal Accuracy (DANVA); Emotional Triangles Task	Social cognition ▪ Children of women with a binging phenotype had higher odds of a poor social communication ▪ Those with a binging and purging phenotype had children with higher odds of having poor social communication DANVA ▪ Children of mothers with a binging and purging phenotype had lower odds of making errors when recognising emotional from high-intensity faces and lower odds of misattributing faces as sad*** Emotional triangle Binging and purging phenotype mothers’ infants’ showed poorer recognition of fear	8/9
5 Cognitive development/Neuropsychological profile Sadeh-Sharvit et al. 2016a	Case–control	Total *n* = 58 Cases *N* = 29; AN = 14, BN = 13, EDNOS = 2, mean age 31 (4.20) Healthy controls *N* = 29, mean age = 33.1 (4.64)	Exposure: self-reported questionnaire (EDI-2) Outcome: Bayley Scales of Infant Development (BSID)	▪ The children of mothers with eating disorders showed delayed mental and psychomotor development ▪ Severity of maternal eating disorder symptoms emerged as a significant predictor of child development, but other maternal psychopathology did not	7/9
6 Cognitive development/Neuropsychological profile Barona et al. 2017	Case–control	Total *n* = 65 Cases *N* = 37; active ED = 18, mean age 30.17 (5.74); past ED = 19, mean age 34.47 (3.96) Healthy controls *N* = 28, mean age = 34.00 (3.34)	Exposure: clinical interview (SCID-I) Outcome: Brazelton Neonatal Behavioural Assessment Scale (NBAS)/Bayley Scales of Infant and Toddler Development (BSID-III)	• Exposed children had poorer neurobehaviour at birth and worse language and motor development at 1 year	8/9
PSYCHOPATHOLOGY					
1 Feeding/Eating/Psychopathology Evans and le Grange 1995*	Case–control	Total *n* = 20 Cases, AN and BN *N* = 10, mean age = 36.0 (7.02) Healthy controls *N* = 10, mean age = 35.4 (4.12)	Exposure: maternal ED (clinical interview?) Outcome: Body Shape Questionnaire; semi-structured interview on feeding and development	▪ Positive correlation found in both case and control groups between mothers’ satisfaction with their body size and their children’s satisfaction with their own weight and shape ▪ Mothers in the clinical group reported experiencing emotional problems when breast feeding their children ▪ Infants in the clinical group were schedule fed—this rigid adherence caused some confusion and anxiety for mothers when their infants displayed signs of hunger outside the recommended feeding times ▪ Half the children of ED mothers were described as displaying difficulties such as hyperactivity, avoidant behaviour, enuresis, insecure attachment, depression, fears, personality problems, stuttering, violent temperament and oppositional defiant behaviour	8/9
2 Psychopathology Barbin et al. 2002	Case–control	Total *n* = 65, mean age = 33.2 (3.1) Cases: *N* = 44 ED group (*N* = 22), depressed group (*N* = 20) Healthy controls *N* = 23	Exposure: maternal ED (self-report questionnaire) Outcome: Child Behaviour Checklist (CBCL); Children’s Eating Behaviour inventory (CEBI); Parent Child Relationship Inventory (PCRI)	▪ No differences in terms of psychological adjustment in children of mothers with ED and controls. ED mothers reported higher number of problems during pregnancy and childbirth ▪ Children of mothers with depression had significantly greater psychological problems compared with children of mothers with ED and controls	8/9
3 Psychopathology/Feeding/Eating Reba-Harrelson et al. 2010*	Cohort	Total *n* = 13,006 Cases *N* = 479, BED = 634, BN = 98, AN = 17 Healthy controls *N* = 12,257	Exposure: maternal ED (self-reported) Outcome: Child Feeding Questionnaire; Child Behaviour Checklist (CBCL); Infant Toddler Social Emotional Assessment (ITSEA)	▪ Mothers with BN and BED reported higher levels of disordered eating behaviours in their children than controls ▪ They also reported higher levels of anxiety symptoms in their children ▪ Mothers with BN reported higher levels of anxiety and OCD symptoms in their children ▪ Maternally reported restrictive feeding was significantly associated with child disordered eating difficulties	8/9
4 Psychopathology Cimino et al. 2013	Case–control	Total *n* = 64, mean age = 33.2 (3.1) Cases *N* = 31; AN = 16, BN = 15 Healthy control *N* = 33	Exposure: maternal ED (clinical interview SCID-I) Outcome: Symptom Checklist-90–Revised (SCL-90-R) Child Behaviour Checklist (CBCL)	Children’s longitudinal profiles ▪ Emotional-adaptive profiles were significantly higher in children in the exposed group on all CBCL dimensions across all three assessment time points Power of maternal EDs on the child’s psychological profile ▪ Mother’s psychoticism score was related to the child’s anxiety/depression and T1 and T2	8/9
5 Psychopathology Micali et al. 2014a	Cohort	Total *n* = 9443 Cases AN = 126, BN = 156 and AN + BN = 62 Healthy controls *N* = 9099 ALSPAC	Exposure: self-reported questionnaire (EDE-Q) Outcome: Development and Well-being Assessment (DAWBA); Strengths and Difficulties Questionnaire (SDQ)	Maternal ED and offspring psychopathology at age 7 ▪ Maternal ED predicted emotional disorders, particularly a strong association between maternal AN and AN + BN for offspring emotional disorders Maternal ED and offspring psychopathology at age 10 ▪ Maternal ED predicted offspring DSM-IV or ICD-10 disorder ▪ Maternal AN and AN + BN strong associated with emotional and anxiety disorders**	7/9
6 Psychopathology Micali et al. 2014b	Cohort	Total *n* = 8622 Cases *N* = 351, AN: *n* = 193, mean age = 29.5 (4.9) BN: *n* = 158, mean age = 28.4 (4.3) AN + BN = 81 Healthy controls *N* = 8271, mean age = 28.7 (4.6) ALSPAC	Exposure: self-reported questionnaire Outcome: Strengths and Difficulties Questionnaire (SDQ); Carey Infant Temperament Scale; Denver Developmental Scale; Life Events Questionnaire; obstetric records	Childhood psychopathology ▪ Gender was predictive of having conduct problems, hyperactivity/inattention but not of emotional problems ▪ Children of women with AN (both genders) and boys of women with BN were more likely to have emotional problems ▪ Children of women with BN were more likely to present with conduct problems ▪ Girls of AN mothers were more likely to have emotional, conduct and hyperactivity problems ▪ Girls of BN mothers were more likely to have hyperactivity/inattention problems ▪ Boys of AN mothers were twice as likely to have emotional problems whereas boys of BN mothers were twice as likely to have emotional and conduct problems	7/9
7 Psychopathology Cimino et al. 2015	Case–control	Total *N* = 251 families, mean age = 33.2 (3.1) Anxiety disorder (*N* = 42) Depressive disorder (*N* = 39) Eating disorder (*N* = 44) Healthy control *N* = 126	Exposure: Maternal ED (clinical interview SCID-I) Outcome: symptoms checklist 90 (SCL-90-R); The Child Behaviour Checklist (CBCL)	▪ Children’s externalising problems tended to increase over time only in the groups of mothers with an ED ▪ Interpersonal sensitivity and psychoticism significantly predicted externalizing problems	8/9
8 Cognitive development/Neuropsychological profile and Temperament Barona et al. 2016*	Cohort	Total n = 48,403 Cases *N* = 2197; AN = 906; BN = 931; AN+BN = 360 Healthy controls *N* = 46,206, mean age = 29.4 (4.16) DNBC	Exposure: self-report questionnaire Outcome: Developmental Milestone Interview; Child Temperament; Looking after Child; SDQ	▪ Girls of women with lifetime AN had higher odds of having emotional problems ▪ Girls of women with lifetime BN of having conduct problems compared with children of healthy women ▪ Boys of women with lifetime AN had higher odd of total, emotional and conduct problems ▪ Boys of women with lifetime BN had higher odds of total, conduct, hyperactivity and peer difficulties compared to children of women without an ED ▪ Boys of women with lifetime AN and BN had higher odds of total, emotional and peer problems compared to children of healthy women	8/9
9 Psychopathology Cimino et al. 2016	Case–control	Total *n* = 408 parents Cases Both parents with BED *N* = 102, children = 51 Only mothers with BED *N* = 104, children = 52 Only fathers with BED *N* = 100, children = 50 Healthy controls *N* = 102 parents, children *N* = 51	Exposure: maternal ED (clinical interview through DSM-5 criteria) Outcome: Scale for the Assessment of Feeding Interactions (SVIA)/Child Behaviour Checklist (CBCL)	▪ The groups with one or both parents diagnosed with BED showed higher scores on the SVIA and on the CBCL internalising and externalising scales, indicating poorer adult–child feeding interactions and higher emotional–behavioural difficulties ▪ A direct influence of parental psychiatric diagnosis on the quality of mother–infant and father–infant interactions was also found, both at T1 and T2	8/9
10 Psychopathology Sadeh-Sharvit et al. 2016b	Case–control	Total *n* = not specified Cases *N* = 29; AN = 14; BN = 13; EDNOS = 2, mean age = 31 (4.20) Healthy controls *N* = not specified, mean age, 33.1 (4.64)	Exposure: self-report questionnaire (EDI-2) Outcome: CBCL/2–3; video recording of mother/child interaction	▪ Mothers with eating disorders were less sensitive to their children, tried to control their children’s behaviours more, and were less happy during mother–child interactions ▪ The children in the maternal eating disorder group were rated as less responsive to their mothers and their mothers also reported more behavioural problems than those in the control group	8/9
11 Psychopathology Cimino et al. 2018	Case–control	Total *n* = 150 families Cases F+/M+ *N* = 50 M−/F+ *N* = 50 F+/M− *N* = 50 Healthy controls *N* = 50	Exposure: maternal ED, clinical interview (SCID-I) Outcome: Italian adaption of Feeding Scale (SVIA)/CBCL	▪ Children with both parents with BED showed the highest affective, anxiety, oppositional/defiant and autism spectrum problems, but no influence of paternal diagnosis was found on the offspring’s psychopathology ▪ Maternal BED had an influence on children’s affective and autism spectrum problems ▪ Diagnosis of BED in both parents had an effect on infants’ affective problems ▪ Paternal BED had an effect on oppositional/defiant problems through the quality of father–infant interactions ▪ Maternal BED had an effect on the offspring’s affective and anxiety problems through the mediation of mother–infant interactions	7/9
TEMPERAMENT					
1 Feeding/Eating/Temperament Agras et al. 1999*	Case–control	Total *n* = 194 Cases *N* = 41, mean age = 32.1 (4.4) AN = 2, BN = 17, BED = 22 Healthy controls *N* = 153, mean age 32.9 (3.8)	Exposure: maternal ED (clinical interview DSM III-R + Eating Disorder Inventory—EDI) Outcome: Infant Feeding Report (IFR); Suckometer; Children’s Behaviour Questionnaire	▪ Female infants of ED mothers sucked more rapidly than other infants but no differences in caloric intake at these feedings ▪ ED mothers bottle fed their daughters for a mean time of 33.2 months compared with infants of NED mothers with 23.6 months ▪ ED mothers considered their female children to have more difficulty in weaning from the bottle (but not from the breast) than NED mothers ▪ Infants of ED mothers were reported to dawdle more while eating compared to children of NED group ▪ ED mothers reported their daughters as vomiting more frequently than their sons (opposite effect for NED mothers) ▪ ED mothers reported higher concern for their daughter’s weight than NED mothers ▪ Significant main effect for the ED group for using food as non-nutritive purposes ▪ Significant effect of ED mothers reporting they fed their children on a less regular schedule than NED mothers ▪ Children of mothers with ED reported as demonstrating more negative affect (sadness, crying, irritability) than children of NED mothers	8/9
2 Feeding/Eating/Temperament Waugh and Bulik 1999*	Case–control	Total *n* = 20 mothers *N* = 10, mean age 30.1 (3.1) Past AN = 6, Past BN = 7 Healthy controls recruited from community *n* = 10, mean age 30.8 (3.6)	Exposure: maternal ED (clinical interview) Outcome: Toddler Temperament Scale (TTS); Mealtime Observation Schedule (MOS); Food Diary	▪ Children of women with eating disorders had significantly lower birth weights and lengths than control children ▪ No differences observed in childhood temperament or mothers’ satisfaction with children’s appearance ▪ Mothers with ED made significantly fewer positive eating comments	8/9
3 Cognitive development/Neuropsychological profile and Temperament Barona et al. 2016*	Cohort	Total *n* = 48,403 Cases *N* = 2197; AN = 906; BN = 931; AN + BN = 360 Healthy controls *N* = 46,206, mean age = 29.4 (4.16)	Exposure: self-report questionnaire Outcome: Developmental Milestone Interview; Child Temperament; Looking after Child; SDQ	▪ Girls of women with lifetime AN had higher odds of having emotional problems ▪ Girls of women with lifetime BN of having conduct problems compared with children of healthy women ▪ Boys of women with lifetime AN had higher odds of total, emotional and conduct problems ▪ Boys of women with lifetime BN had higher odds of total, conduct, hyperactivity and peer difficulties compared to children of women without an ED ▪ Boys of women with lifetime AN and BN had higher odds of total, emotional and peer problems compared to children of healthy women	8/9
4 Temperament Zerwas et al. 2012	Cohort	Total *n* = 48,964 Cases *N* = 3013, AN: *n* = 44 mean age = 26.5 (4.7) BN: *n* = 436 mean age = 29.6 (4.7) BED: *n* = 2475, mean age = 30.1 (4.6) EDNOS: *n* = 58 mean age = 27.8 (5.2) Healthy controls *N* = 45,964	Exposure: maternal ED (self-report questionnaire) Outcome: Fussy/Difficult Subscale–Infant Characteristics Questionnaire	▪ Mothers with an ED had greater odds of reporting more difficult infant temperament ratings ▪ Women with AN and EDNOS-P were 2.3–2.8 times more likely to rate their children’s temperament as difficult	9/9

AN, anorexia nervosa; BED, binge-eating disorder; BN, bulimia nervosa; ED, eating disorder; SCID, Structured Clinical Interview for DSM-IV

*Studies fitting more than one domain

For each study, the following data were extracted when available: demographic information, including participant characteristics (sample size ad mean age), ED type (AN, BN, BED), ED status (actual vs. recovered); measures used to certain exposure and outcome. The identified studies investigated a number of different child outcomes. After discussion between authors (M.B., M.G.M., N.M.), four domains were discussed: feeding/eating, neuropsychological profile/cognitive development, psychopathology and temperament. However, a range of studies investigated more than one outcome and were included in more than one domain (Table 1).

## Results

### Search findings

**Fig. 1:**
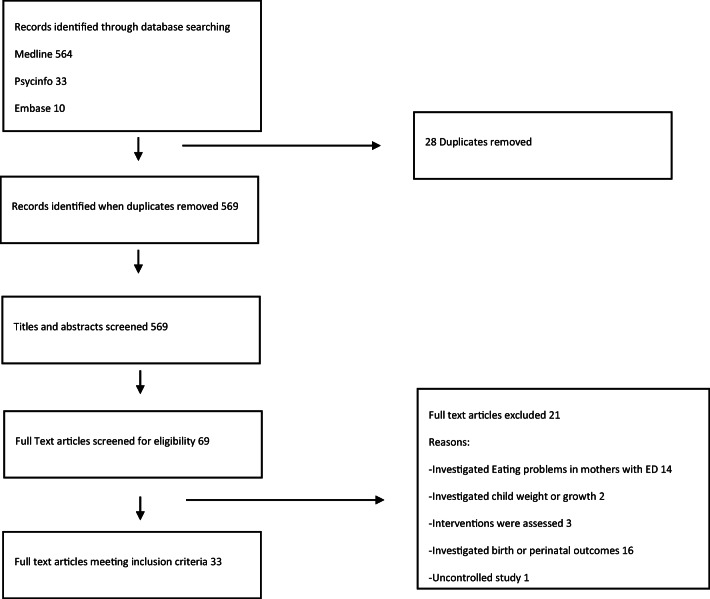
PRISMA flow diagram showing study selection process

A PRISMA flow diagram presents all phases of the review (Fig. 1) (Moher et al. 2009).

Most studies used case–control (*n* = 17) and cohort design (*n* = 16) and one early study used an uncontrolled design. The majority of studies were published within the last decade (*n* = 25) with 59% published within the last 5 years (2013–2018).

Studies included measured various aspects of child development: feeding/eating (*n* = 17), cognitive development/neuropsychological profile (*n* = 6), psychopathology (*n* = 12) and infant temperament (*n* = 4). Five studies fit more than one domain.

Characteristics of the 34 articles included in the systematic review are shown in Table 1.

### Quality assessment

The research design and content of studies was well executed with over 90% rated as strong. The four studies rated as moderate were due to (1) poor representativeness of cases, poorly defined controls and ascertainment of exposure; (2) lack of comparability of cases and controls on the basis of design or analysis (Blissett and Meyer 2006); (3) one study being uncontrolled (Hodes et al. 1997); and (4) no description of control group, no mention of history of outcome (Saltzman et al. 2016). It is worth noting that sample size was not accounted for as some studies rated as strong included small sample sizes of less than 10 (Evans and le Grange 1995). Therefore, a strong and positive overall assessment should be interpreted with caution.

### Study results

#### Feeding outcomes (*n* = 17)

Given its relevance, the impact of maternal ED on children’s feeding and mealtimes is an area that has received considerable attention. Feeding the infant is a crucial task of parenting not only because it can absorb considerable part of the day in the early stages of life but also because it is one of the most important means of communication between mother and child (Silva et al. 2016).

Review of the present studies showed that mothers with ED might exhibit problems in feeding behaviours with their offspring starting from breast feeding. A number of population-based studies investigated the impact of ED on breast feeding, yielding discrepant findings (Micali et al. 2011, 2009). A large population-based study showed that women with a history of ED were more likely to start breast feeding and less likely to stop during the first year of the infant life. More specifically, mothers with BN were more likely to continue breast feeding and these differences persisted even after adjusting for confounding factors (Micali et al. 2009). A similar study found that women with ED started breast feeding their infants as often as controls; however, they were more likely to interrupt early (Torgersen et al. 2010). Conversely, Nguyen and colleagues found that mothers with a history of ED were slightly less likely to initiate breast feeding, although no longer significant after adjustment (socio-demographics, body mass index (BMI), maternal psychiatric symptoms) (Nguyen et al. 2017).

Although feeding difficulties are common in early stages of the infant’s life, mothers with AN reported increased feeding problems including exhaustion during feeding, slow feeding and no established feeding routine (Micali et al. 2011, 2009). On the other hand, infants of women with BN had higher levels of refusal to take solids in comparison to controls (Micali et al. 2009). Blisset and Meyer found similar results with some gender specificity, showing that maternal eating psychopathology predicted food refusal in girls but not boys. Symptoms of low maternal depression combined with high bulimic scores were significantly associated with food refusal in girls (Blissett and Meyer 2006). Conversely, Whelan and Cooper did not find the gender of the child to be related to maternal ED history, nor did it moderate the relationship between feeding difficulties and maternal ED (Whelan and Cooper 2000).

There is a well-known relationship between specific parenting styles in women with EDs and feeding problems in their offspring. An early study found that negative expressed emotions and more intrusive behaviours were more frequent among mothers with ED compared to controls during both mealtime and play (Stein et al. 1994). Mealtime interaction in offspring (ages 1–4) and mothers with ED compared to controls was also investigated by Waugh and Bulik (1999) who found that control mothers made more positive eating comments while mothers with ED tended to be more negative.

Differences between ED diagnoses and their impact on infant feeding problems are worth noting. Saltzman and colleagues found that maternal BED predicted use of more nonresponsive feeding practices (i.e. emotion regulation, restriction for health, pressure to eat and food as reward), indirectly through more distress responses to children’s negative emotions. Maternal BED was associated with greater use of distress responses, which indirectly predicted higher child BMI percentile through food as reward feeding practices (Saltzman et al. 2016). Also, mothers with BN or BED were more likely to report higher levels of restrictive feeding styles compared to controls (Reba-Harrelson et al. 2010), and mothers with a history of AN were more likely to use less pressuring feeding strategies compared to controls (de Barse et al. 2015).

Maternal ED can also influence nutritional intake, dietary patterns and diet quality in infants.

Easter and colleagues found that children of mothers with ED reported higher scores on the ‘health conscious/vegetarian’ dietary pattern (diet high in vegetarian foods, nuts, salad, rice, pasta and fruits) across all four time periods (ages 3, 4, 7 and 9) when compared with controls (Easter et al. 2013). Furthermore, they showed less adherence to the ‘traditional’ dietary pattern across all four time points. Energy intake was higher in the BN and AN + BN group, and this intake increased significantly over time for children of mothers with BN (Easter et al. 2013). In another study, Torgersen et al. (2015) found that mothers with BN and BED were less likely to use ‘homemade traditional food’ than ‘commercial jarred baby food’ when compared with mothers without ED; however, in women with BED, the associations were significant only before controlling for relevant confounders (Torgersen et al. 2015). Nguyen and colleagues found that children of mothers with life-time ED had higher diet quality at 1 year (Nguyen et al. 2017). Mothers with histories of ED may also be more likely to take ‘special approaches’ to feeding their children, such as limiting the amount of processed foods or eliminating other types or classes of foods (Hoffman et al. 2014).

Body image distortion, core symptoms of both AN and BN can also have an impact on feeding practices. In a recent study conducted by our team, we found that women with both past and current ED reported higher concerns about their infant being/becoming overweight compared with controls, respectively, at 8 weeks and 6 months and 6 months only postnatally. Also, women with past ED showed less awareness of infant hunger and satiety cues compared with HC at 8 weeks (Martini et al. 2018).

#### Cognitive development and neuropsychological profile (*n* = 6)

There is a growing evidence to suggest that ED are characterised by a specific neurocognitive profile including alterations in attention, visuospatial ability, memory, social cognition and executive functions, encompassing set shifting and central coherence (Giombini et al. 2018; Lang et al. 2014; Westwood et al. 2016). These profiles observed during active illness were thought to be a complex interplay between risk and protective factors, given the genetic profile and the drive to high educational achievement and perfectionism, common features of ED profile (Bardone-Cone et al. 2010). Also, these might be intermediate phenotypes, an early risk marker that are thought to lie between genetic/biological risk and actual phenotypical manifestations (Micali and Dahlgren 2016).

Research on neuropsychological profiles in children of women with ED aimed mainly at understanding both the impact of maternal ED on their children and to obtain a better understanding of intermediate endophenotypes of ED.

In the study carried out by Kothari et al. (2013), the investigation of neuropsychological profiles showed that children of mothers with AN had high full-scale and performance IQ, increased working memory (WM) capacity, better visuospatial functioning and decreased attentional control when compared to controls (Kothari et al. 2013). Another study investigating the early cognitive development (18 months and 4 years) in a sub-set of these children showed that children of women with lifetime AN had difficulties with social understanding, poorer motor skills, planning and abstract reasoning (Kothari et al. 2014). In a recent longitudinal study of children of mothers with ED, we recently found that infants of mothers with ED had poorer language and motor development compared to control mothers. Interestingly, after studying differential outcomes based on active versus past ED, we found that child cognitive difficulties were associated both with maternal active ED and past ED (Barona et al. 2017). Overall, these findings highlight early developmental difficulties in motor and cognitive development in offspring of ED mothers.

The study of later cognitive characteristics suggests a more specific pattern of strengths and difficulties across several domains of cognition, for example, higher IQ, better visuospatial performance and WM in children of mothers with lifetime AN, and poorer visuospatial functioning in children of mothers with lifetime BN (Barona et al. 2017).

Only one study to date has investigated social cognition in children of mothers with ED in mid-childhood and early adolescence, with some evidence of differences between at-risk offspring and controls. In particular, differential facial emotion processing, poorer recognition of fear, and higher scores on the social communication disorders checklist were present in children of mothers with binge eating and purging behaviours, the latter also present in children of mothers with lifetime BED (Kothari et al. 2015).

Finally, in a small longitudinal cohort study, Koubaa and colleagues found that children born to mothers with AN or BN had significantly higher Five to Fifteen (validated parent questionnaire assessing neurocognitive development) scores than controls reflecting difficulties in language and social skills (Koubaa et al. 2013).

#### Psychopathology (*N* = 12)

There is strong evidence that parental mental illness is associated with psychopathology in offspring, establishing that children of parents with psychiatric disorders are at higher risk of developing psychopathology themselves (Rasic et al. 2013). Research has also shown that familiar risk is only partly diagnosis-specific meaning that children might develop the same parental diagnosis (homotypic transmission) but may be also at risk for a range of other psychiatric disorders (heterotypic transmission) (Siegenthaler et al. 2012). However, the effect of maternal ED on their offspring psychopathology has received less attention.

Of the studies in this domain, five specifically investigated psychological/emotional development (Barbin et al. 2002; Cimino et al. 2016, 2013, 2015; Sadeh-Sharvit et al. 2016b) and seven explored general psychopathology (Barona et al. 2016; Cimino et al. 2018; Evans and le Grange 1995; Hodes et al. 1997; Micali et al. 2014a, 2014b; Reba-Harrelson et al. 2010).

A large longitudinal cohort study identified a higher risk for emotional and behavioural disorders in offspring of ED mothers at an early age (3.5 years). Specifically, the authors identified associations between maternal ED type (AN vs. BN) and child psychopathology (emotional vs. behavioural problems), as well as gender differences (Micali et al. 2014b). Emotional difficulties were found in both girls and boys of mothers with AN, while more hyperactivity and peer difficulties were found in both girls and boys of mothers with BN. This study also aimed to understand risk mechanisms and highlighted a mediating role for maternal anxiety and depression in the postpartum. These findings were confirmed and extended in a later study when the same children were aged 7, 10 and 13, where maternal EDs (both AN and BN) strongly predicted emotional and anxiety disorders (Micali et al. 2014a). A recent longitudinal study showed that maternal ED was associated with childhood psychopathology in girls and boys at age 7, both emotional and behavioural problems. Disorder and gender-specific findings across studies point to an effect of maternal lifetime AN on emotional disorders, in particular anxiety, across ages; while maternal BN seems to be associated with hyperactivity, conduct and peer problems (Barona et al. 2016).

These findings support those of case–control studies. In two longitudinal studies using the Child Behaviour Checklist (CBCL), the authors found that children of mothers with ED reported higher externalising and internalising scores from the CBCL compared to controls (Cimino et al. 2015, 2018). The emotional-adaptive profiles of children in the clinical group of mothers with ED scored significantly higher in all dimensions of the CBCL (Cimino et al. 2013). More specifically, externalising problems increased over time (Cimino et al. 2015).

The same authors carried out other two longitudinal studies aimed at investigating the emotional and behavioural profile of children of one or both parents with BED and adult–child feeding interaction (Cimino et al. 2016, 2018). They found that the children with one or both parents diagnosed with BED had higher scores on the internalising and externalising scales, and higher levels of emotional and behavioural difficulties (Cimino et al. 2016). Furthermore, children with both parents with BED showed the highest affective, anxiety, oppositional/defiant, and autism spectrum problems versus children of families with only one BED diagnosed father. Maternal BED was associated with children’s affective and autism spectrum problems, and diagnosis of BED in both parents had an effect on infants’ affective problems. Paternal BED had an effect on oppositional/defiant problems through the quality of father–infant interactions, and maternal BED had an effect on the offspring’s affective and anxiety problems through the mediation of mother–infant interactions (Cimino et al. 2018).

On the contrary, Barbin et al. did not find any difference in terms of psychological adjustment on both subscales of internalising and externalising in children of mothers with ED compared to healthy controls (Barbin et al. 2002).

In two early small studies, authors found that half of children of mothers with ED displayed psychiatric problems such hyperactivity, avoidant behaviour, enuresis, insecure attachment, depression, fears, personality problems, stuttering, violent temperament and oppositional defiant behaviour (Evans and le Grange 1995) and OCD (Hodes et al. 1997), respectively.

The latter association has also been found in a more recent large cohort study conducted by Reba-Harrelson and colleagues. The authors found that children of mothers with binge BED or BN were significantly more likely to exhibit symptoms of anxiety compared to children of control group mothers. Children of mothers with BN were also significantly more likely to exhibit obsessive-compulsive symptoms than children of non-eating-disordered mothers. All those with AN, BN or BED were significantly more likely to have children who exhibited symptoms of depression (Reba-Harrelson et al. 2010).

Likewise in a small recent study, mothers with ED reported higher levels of behavioural problems in their children compared to control mothers (Sadeh-Sharvit et al. 2016b).

#### Temperament (*N* = 4)

Temperament is defined as early appearing, biologically rooted, basic personality dimension (Bates et al. 1995). Although temperament is said to be a biological tendency within each child, environmental contexts, especially the social context, interrelate with the genetic tendencies of each child (Shiner et al. 2012).

Mothers with EDs have been shown to be more likely to describe their infant as having a difficult temperament. Compared to women with no history or current EDs, they tend to rate their children as having high levels of difficult temperament (Zerwas et al. 2012) and perceive them as having greater negative affect, that is, demonstrating more sadness, irritability and crying compared to controls (Agras et al. 1999). In a more recent large longitudinal study, we found that mothers with ED were more likely to perceive their children as having a ‘difficult temperament’ characterised by the child being less happy and active than other children their age, more restless and as having more tantrums and as being less cautious and guarded than other children their age (Barona et al. 2016). On the contrary in an early study, the authors did not observe any difference in childhood temperament between children born from ED mothers and controls (Waugh and Bulik 1999).

## Discussion

The literature regarding the impact of maternal ED on child development has expanded in the last decade. Albeit earlier studies focusing on small clinical samples, larger population-based samples have recently increased our knowledge on the topic.

Overall, the literature suggests that maternal ED impact on child cognitive, psychological and feeding and eating development.

Regarding feeding and eating, research shows that children of mothers with ED tend to experience more difficulties in feeding their children both in infancy and childhood. Although studies on breast feeding yield mixed results, women with ED often report difficulties with this. Throughout toddler years, difficulties such as slow feeding, small quantities feeding and not having established feeding routines emerged as common among ED mothers. Restricting parental feeding styles, negative expressed emotions and intrusive behaviours also appeared to be more frequent in ED mothers compared to controls. The most recent findings (Martini et al. 2018; Saltzman et al. 2016) are in line with earlier research (Stein et al. 1994). To date, it is not possible to disentangle the influence of genetic predisposition versus environmental factors, though likely both have a role. Future research investigating genotype–environment interface is warranted to provide further insight.

Although the current literature is less prominent, available findings shows that a maternal lifetime history of ED may have a negative impact also on children’s cognitive, psychological development and psychopathology.

Interestingly, recent studies have identified shared genetic risk between ED and other psychiatric disorders, i.e. schizophrenia and BED (Solmi et al. 2019) and AN and obsessive compulsive disorder (OCD) but also other positive genetic correlations between AN and schizophrenia and AN and neuroticism (Duncan et al. 2017; Yilmaz et al. 2018). These findings suggest that the genetic risk transmitted will impact on the behavioural, physiological and medical phenotypes expressed in children. Therefore, we might assume that children of AN women have a higher genetic risk of developing a wide range of phenotypes and psychiatric disorders such as psychosis, neuroticism, educational attainment but also lower BMI and lower risk of diabetes. Less is known on cognition and neuropsychological characteristics in offspring of ED mothers prior to the onset of psychopathology, and these might be an important focus of study in at-risk offspring, helping future prevention and early interventions.

### Strengths and limitations

This systematic review has several strengths. First, it is the first systematic review summarising the existing findings from 1980 until 2018 on the impact of maternal ED on child development, and its findings can be valuable in deriving future hypothesis.

Second, the majority of studies relied on community-based or register-based samples rather than clinical samples; therefore, selection bias in the original studies is likely to be small.

Moreover, if an association between maternal ED and child development has been established in population-based studies, which are more likely to include less severe case of ED, our results are liable to be an underestimation of the effect.

However, some limitations should also have to be taken into account. Some of the studies reviewed above are single findings and need replication. Also, the vast majority of the studies explored mother–child dyad and did not involve the fathers. Although ED have a higher incidence in women than in men, gender ration is less skewed in BED than BN and AN (Hudson et al. 2007) and paternal ED can also have an impact on child development.

Studies showed a high degree of heterogeneity in the way the ED diagnosis is established, ranging from self-reported questionnaires (used in cohort studies such as ALSPAC (Easter et al. 2013; Micali et al. 2009, 2011, 2014a, 2014b, ), DNBC (Barona et al. 2016), Generation R study (de Barse et al. 2015; Nguyen et al. 2017), MoBa (Torgersen et al. 2015)) to structured interviews using both the Diagnostic and Statistical Manual (Agras et al. 1999; Cimino et al. 2016; Evans and le Grange 1995; Stein 1999; Waugh and Bulik 1999; Whelan and Cooper 2000) and SCID I (Barona et al. 2017; Cimino et al. 2015, 2018; Hoffman et al. 2014; Kothari et al. 2015; Martini et al. 2018).

It is important to point out that parental psychopathology does not always impact on child development in a negative way. This might be due to numerous factors including protective family network, child resilience, early access to mental health services, parenting abilities etc.

Future research should focus on the protective factors that moderate the relationship between maternal ED and adverse child outcomes leading to a healthy development of the child. This would shed light into mechanisms that could be potentially directed for intervention. For example, further research could introduce fathers and explore their roles in the family dynamics and in ameliorating, exacerbating or acting independently of the possible influence of a mother with ED.

## Conclusion

In summary, maternal ED have an impact on child psychological, cognitive and eating development, and might affect the development of ED in the offspring. Despite this evidence, little is known on how genetic and environmental factors interplay and affect risk. As maternal psychopathology not always negatively affect child development, future research should focus on resilience and on which protective factors have impact on positive outcome. These factors can be then used as therapeutic and preventative targets.
